# Simultaneous versus Sequential Accelerated Corneal Collagen Cross-Linking and Wave Front Guided PRK for Treatment of Keratoconus: Objective and Subjective Evaluation

**DOI:** 10.1155/2016/2927546

**Published:** 2016-12-29

**Authors:** Waleed Ali Abou Samra, Dalia Sabry El Emam, Rania Kamel Farag, Hossam Youssef Abouelkheir

**Affiliations:** Mansoura Ophthalmology Center, Mansoura University, El Gomhoriah Street, Mansoura, Egypt

## Abstract

*Aim*. To compare objective and subjective outcome after simultaneous wave front guided (WFG) PRK and accelerated corneal cross-linking (CXL) in patients with progressive keratoconus versus sequential WFG PRK 6 months after CXL.* Methods*. 62 eyes with progressive keratoconus were divided into two groups; the first including 30 eyes underwent simultaneous WFG PRK with accelerated CXL. The second including 32 eyes underwent subsequent WFG PRK performed 6 months later after accelerated CXL. Visual, refractive, topographic, and aberrometric data were determined preoperatively and during 1-year follow-up period and the results compared in between the 2 studied groups.* Results*. All evaluated visual, refractive, and aberrometric parameters demonstrated highly significant improvement in both studied groups (all *P* < 0.001). A significant improvement was observed in keratometric and *Q* values. The improvement in all parameters was stable till the end of follow-up. Likewise, no significant difference was determined in between the 2 groups in any of recorded parameters. Subjective data revealed similarly significant improvement in both groups.* Conclusions*. WFG PRK and accelerated CXL is an effective and safe option to improve the vision in mild to moderate keratoconus. In one-year follow-up, there is no statistically significant difference between the simultaneous and sequential procedure.

## 1. Introduction

Keratoconus (KC) is a bilateral, nonsymmetric, noninflammatory progressive corneal dystrophy that results in biomechanical weakening and progressive steepening [1].

Although corneal cross-linking (CXL) using riboflavin and ultraviolet A has been used to stabilize the cornea with progressive keratoconus or ectatic corneal disorders, many studies demonstrated that combining surface ablation with CXL offers keratoconic patients both stability and functional vision with improvements in uncorrected distance acuity, best distance acuity, and topographic irregularity, even if the surgical goal is not a refractive end point [2–4].

However, most of studies demonstrated the visual and topographical changes in simultaneous (same day) photo refractive keratotomy (PRK) with CXL [5–9]. To the best of our knowledge only one study compared retrospectively objective outcomes, safety, and efficacy of simultaneous versus sequential standard CXL and topo guided PRK (t-PRK) for treatment of keratoconus [10].

The purpose of this prospective study is to compare and follow up the topographic, aberrometric, and refractive results for one year in keratoconic patients treated with simultaneous wave front guided (WFG) photorefractive keratotomy (PRK) and accelerated CXL with those of patients treated with sequential WFG PRK 6 months after accelerated CXL. Moreover, the subjective findings of both techniques were recorded and compared.

## 2. Subjects and Methods

### 2.1. Patients

In this prospective study, patients aged 21 years or more with grade I and II progressive KC (according to Amsler-Krumeich classification) were collected from outpatient clinics of Mansoura ophthalmic center, Mansoura University. The diagnosis of keratoconus was based on corneal Pentacam and slit-lamp observation. Inclusion criteria were the existence of progressive KC with a clear cornea in the visual axis (absence of scar and Vogt striae) with average keratometric reading (*K*) less than 53 diopters (D) and minimal corneal thickness more than 420 um (including the epithelium thickness). Keratoconus was progressive if maximum keratometry (*K*max) of the cornea changed more than 1.00 D on corneal topography, the cylinder increased more than 0.50 D, or the corneal pachymetry decreased more than 20 microns during the 6-month follow-up.

Manifest spherical equivalent refraction of the studied eyes was ≤4 D with best-corrected visual acuity (BCVA) 0.05 LogMAR or better. The expected residual corneal stromal bed thickness should not be less than 350 um (without including the epithelium thickness) after maximum ablation of 50 um at the corneal center. However, we attempted correction of 75%–80% of the measured sphere and cylinder if the calculated ablation exceeded 50 um.

Exclusion criteria were dry eye syndrome, a history of herpetic eye disease, history of laser-assisted in situ keratomileusis, pregnancy or lactation during the course of the study, active anterior and posterior segment pathologies, autoimmune disease, and delayed epithelial healing. The patients with very thin corneas, below 400 um (including the epithelium thickness), corneal scarring or opacification within the pupillary area preventing a reliable acquisition with the aberrometer, or very poor corrected distance visual acuity (CDVA) were not included in the study. Contact lens wearers were instructed to discontinue contact lens use for a minimum of 2 weeks before the preoperative eye examination for soft contact lenses and 4 weeks for hard contact lenses.

The study was reviewed and approved by the institutional review board (IRB) of Faculty of Medicine, Mansoura University. The study and the surgical procedure were first explained to the subjects eligible for intervention with a signed consent from every patient following the Declaration of Helsinki.

Using computer-generated random numbers, the patients enrolled in the study were divided into 2 groups: one underwent simultaneous (same day) WFG PRK with CXL and another underwent CXL followed by WFG PRK 6 months later provided by stability of the case following CXL procedure [±0.5 D of change in spherical equivalent (SE)] in 3 consecutive monthly visits combined with keratometric stability (no increase of the cone apex keratometry of ≥0.75 D) in the last 6 months of follow-up.

### 2.2. Preoperative and Postoperative Examination

Preoperatively, all patients had a full ophthalmological examination including uncorrected distance visual acuity (UDVA) and corrected distance visual acuity (CDVA), manifest and cycloplegic refraction, slit-lamp evaluation, Goldman applanation tonometry, and fundoscopy. Moreover, anterior segment imaging using the Pentacam-HR system (Oculus Optikgeraete GmbH, Wetzlar, Germany), contrast sensitivity and glare tests (Mesotest II, Oculus, Germany), and ocular aberrometry (Zywave II, Bausch and Lomb, Munich, Germany) were performed.

Using the Pentacam topography system, the following topographic and pachymetric parameters were recorded and evaluated: flattest keratometric reading (*K*1), steepest keratometric reading (*K*2), maximum keratometry (*K*max), corneal asphericity (*Q* value), minimum corneal thickness (MCT), index of surface variation (ISV), index of vertical asymmetry (IVA), index of height asymmetry (IHA), minimum radius of curvature (*R*min), and keratoconus index (KI). With the high-resolution aberrometer, total root mean square (RMS) and higher order aberration (HOA) RMS were recorded and evaluated for a 6 mm pupil.

Postoperatively, patients were examined the third day after any surgery and at 1 week postoperatively to assess the status of corneal epithelial healing. If complete epithelialization was present, the therapeutic contact lens fitted after the surgery was removed. Afterward, the patients were examined 3, 6, and 12 months after surgery in the first group. Patients enrolled in the 2nd group were followed up for 6 months after CXL and then PRK was done after which the patients were examined 3, 6, and 12 months after the PRK procedure. UDVA and CDVA testing, manifest refraction including the mean refractive spherical equivalent (MRSE), corneal topography, ocular aberrometry, mesopic vision, and biomicroscopic examination were performed in each visit.

### 2.3. Contrast and Glare Sensitivity Test

The test was carried out with Mesotest II (Oculus, Germany), which consists of Landolt rings of different contrast levels presented in front of a low-brightness backdrop. There are 4 contrast levels: 1 : 23/1 : 5/1 : 2.7/1 : 2 which represent the ratio between light intensity of the optotypes and the backdrop. There are 8 tests (4 without and 4 with glare). Test 1, with contrast level 1 : 23, is the most easily recognized. For statistical purposes, each level of the contrast or the glare test was given a score staring from 25% of 1 : 23 level to 100 of 1 : 2 level.

### 2.4. Subjective Analysis

Subjective assessment in the study was done by asking the patients to fill out a questionnaire just before performance of the procedure and 12 months later. It included clarity of the vision, patient satisfaction, visual disturbance items (glare and halo), and far spectacle dependence.

Clarity of vision and patient satisfaction were scored on a scale of 1 (none) through 5 (excellent) and visual symptoms were scored on a scale of 1 (none) through 5 (severe) while spectacle dependence was ranked by asking how often the patients used spectacles for distance and near vision: never = 1, rarely = 2, on occasion = 3, often = 4, always = 5. The data of subjective outcomes were presented as the mean subjective visual score for each of the queried parameters.

### 2.5. Surgical Technique of Simultaneous PRK and CXL

Under topical anesthesia, the central 9 mm of the epithelium was removed using PTK mode of the TENEO 317 Excimer LASER (TECHNOLAS Perfect Vision GmbH, A Bausch & Lomb Company, Munich, Germany).

WFG PRK ablation was then applied according to the measurements obtained with the Zywave II aberrometer (TECHNOLAS Perfect Vision). An adjustment to the laser profile was applied to ensure minimal tissue removal (not to exceed 50 *μ*m) by reducing the sphere component without changing the cylinder and/or reducing the effective optical zone diameter. After PRK ablation, a 6 mm cellulose disc soaked in mitomycin C 0.02% solution was applied over the ablated tissue for 20 seconds followed by irrigation with 30 ml of chilled balanced salt solution.

Accelerated CXL was then performed by soaking the exposed stroma with isotonic riboflavin 0.1% in 20% dextran (Medio Cross Isotonic Solution; Medio-Haus Medizinprodukte GmbH, Kiel, Germany) every 2 minutes for 30 minutes. Riboflavin absorption throughout the corneal stroma and anterior chamber was confirmed by slit-lamp examination. Accelerated CXL was performed using 5 minutes of exposure of continuous ultraviolet A (UVA) 365 nm light (Peschke GmbH CCL-365-18; Peschke Meditrade GmbH) at an irradiance of 18.0 mW/cm^2^ (5.4 J/cm^2^ fluence). During UVA exposure, the cornea was kept wet with a drop of distilled water for injection every minute and riboflavin was not applied during the 5-minute treatment time. All patients were treated postoperatively with topical moxifloxacin 0.5% (Vigamox, Alcon Laboratories) applied 4 times daily up to 2 weeks. A combination of tobramycin 3 mg/mL with dexamethasone 1 mg/mL (Tobradex; Alcon Laboratories) was applied 4 times daily for 2 weeks with gradual tapering over further 2 weeks. Preservative-free tear substitute was applied every 2 hours for 2 weeks or more (Refresh Plus; Allergan, Inc., Irvine, TX). A bandage soft contact lens (Cooper Vision, Scottsville, NY) was placed. The contact lens was removed after epithelial healing (3–5 days postoperatively).

### 2.6. Surgical Technique of Sequential PRK and CXL

Accelerated CXL was performed as described above with the same postoperative regimen. WFG PRK was done 6 months later in the same manner described before with similar postoperative follow-up and regimen of simultaneous procedure.

### 2.7. Statistical Analysis

Statistical analysis was performed using SPSS software for Windows version 19.0 (SPSS Inc., Chicago, IL). The recorded data were reported as mean ± standard deviation. Repeated-measures ANOVA with Bonferroni corrected post hoc testing was used to analyze the changes from baseline values (preprocedure levels) to 6 and 12 months' values and between 6 and 12 months' values. Paired *t*-test was used to compare the pre- and postoperative values of subjective outcome. The unpaired *t*-test was performed to compare outcome data and the changes over 12-month period between the 2 studied groups. The level of statistical significance was set at *P* < 0.05. Visual acuity measurements were calculated as logMAR.

## 3. Results

In total, 30 eyes of 30 patients (14 male and 16 female) and 32 eyes of 32 patients (15 male and 17 female) were included in the simultaneous group (1st group) and sequential group (2nd group), respectively. The mean patient age in the 1st group was 26.3 ± 4.2 years and 24.8 ± 5.5 years in the 2nd group years (*P* = 0.33). Demographic data of the studied patients were summarized in Table 1.

### 3.1. Visual and Refractive Outcomes

All of the measured preoperative parameters of the sequential group did not change significantly 6 months after CXL except *K*max that decreased significantly from 48.3 ± 5.4 D to 46.1 ± 5.6 D (*P* = 0.05) (Table 2).

Table 3 summarizes the preoperative and postoperative visual and refractive outcomes of both studied groups obtained in the current study preoperatively and 6 and 12 months postoperatively with corresponding *P* values. As shown, all evaluated visual and refractive parameters demonstrated highly significant improvement after the surgery in both studied groups (all *P* < 0.001) at 6 months after surgery. The improvement in such parameters was found to plateau after 6 months till the end of follow-up, so no significant changes occurred from 6 to 12 months following the full procedure in both groups. Although the results revealed better visual and refractive outcome in the sequential group, they did not reach a statistically significant level in any of recorded parameters. Moreover, comparison of changes occurring in each group from baseline values to last follow-up showed no statistically significant difference in the mean of any studied parameter between the 2 groups (Table 6).

### 3.2. Corneal Morphologic Changes

Table 4 summarizes the corneal morphologic changes occurring after surgery in the studied eyes. As shown, a significant decrease was observed in *K*1, *K*2, *K*max, and *Q* values at 6 months after surgery (*P* < 0.001), with no any additional significant reduction of these parameters during the remaining follow-up. A pachymetric reduction was observed 6 months postoperatively (*P* < 0.001), with minimal increase of the MCT during the rest of the follow-up. Although no significant changes were detected in IVA and IHA, significant changes were detected in the ISV (*P* = 0.003 and *P* = 0.004 in both groups, respectively), *R* min (*P* = 0.032 and *P* = 0.049 in both groups, respectively), and KI (*P* = 0.008 and *P* = 0.006 in both groups, respectively) at 6 months postoperatively with plateau level till the end of follow-up. Independent *t*-test showed no significant difference in between both simultaneous and sequential group in any of recorded topographic parameters preoperatively or postoperatively. Likewise, comparison of changes occurring in each group during follow-up period revealed no significant difference between the 2 groups (Table 6).

### 3.3. Ocular Aberrometric and Mesopic Vision Changes

For both groups the total RMS and HOA RMS were reduced significantly at 6 months after surgery (*P* < 0.001), with additional minimal nonsignificant reduction during the rest of the follow-up. Similarly values of contrast sensitivity and glare tests increased significantly 6 months postoperatively till end of follow-up (*P* < 0.001). Independent *t*-test demonstrated no statistically significant difference in between the 2 studied groups in any of recorded data (Table 5).

### 3.4. Subjective Outcome

Subjective parameters and measures of patient satisfaction were demonstrated in Table 7. They showed statistically significant improvement in all measured parameters. However, no significant difference in between the groups was detected by independent *t*-test.

## 4. Discussion

CXL increases the degree of interfibrillar linkages, thereby improving the biomechanical strength of the cornea. Several studies have approved the successful cessation of keratoconus progression after CXL [11–15].

Previous studies have reported that simultaneous use of t-PRK and CXL is safe and effective and provides good functional vision in eyes with keratoconus [5, 16, 17]. Initial investigations by Kanellopoulos revealed that simultaneous treatment (t-PRK followed immediately by CXL) produces superior outcomes when compared with sequential treatment [10].

However, safety and efficacy of simultaneous versus sequential PRK after CXL are still debatable as CXL alone may or may not improve the refractive status of the eye; thus it is logical to wait till refractive stability following CXL procedure [18]. In another point of view, performing both procedures at the same time appeared to minimize the potential superficial stromal scarring and corneal haze resulting from PRK. Moreover, the potential benefit of CXL can be decreased when PRK is performed after the CXL procedure due to removal of some cross-linked anterior cornea [10]. Thus it was interesting for us to address the evolution of the visual, refractive, topographical, and subjective changes through the first year of follow-up after simultaneous versus sequential WFG PRK and CXL in patients with keratoconus.

### 4.1. Visual and Refractive Outcomes

In a comparative study of sequential versus simultaneous t-PRK and CXL in 325 keratoconus eyes with a mean follow-up of 36 months, Kanellopoulos found more improvement in UDVA and CDVA and a greater mean reduction in MRSE in a simultaneous group as compared with a sequential one [10]. Similarly, Kymionis et al. in 2011 reported encouraging results in 26 patients (31 eyes) with simultaneous t-PRK and CXL for keratoconus. The obtained improvements remained stable at a mean follow-up of nearly 20 months [17].

In the current study, our results using WFG PRK do not differ consistently from those using topography-guided ablation profiles. This is possibly due to the capability of the Zywave II aberrometer device for measuring ocular aberrations that allows good characterization of large amounts of high-order aberrations [19]. All evaluated visual and refractive parameters demonstrated highly significant improvement after the surgery in both studied groups (all *P* < 0.001) at 6 months after surgery with no significant changes till the end of follow-up indicating plateauing and stabilization of outcome (Table 3). On the other hand, in one study carried in early cases of keratoconus using non-topo guided PRK with CXL, the authors demonstrated significant improvement in the UDVA, spherical equivalent, and cylinder while the CDVA remained stable [20]. The significant visual improvement obtained in our study in both UDVA and CDVA was due to the reduction of the spherocylindrical error and the minimization of HOAs. The MRSE was reduced from a mean preoperative value of −3.7 ± 2.4 and −3.8 ± 2.6 in simultaneous and sequential groups, respectively, to a mean postoperative value of −1.3 ± 0.79 and −1.00 ± 0.68 at the last follow-up visit. Similar results were recorded by Shaheen et al. using sequential WFG PRK at least one year after CXL in keratoconus [18]. Kymionis et al. [4] and Alessio et al. [21] reported in eyes with keratoconus receiving simultaneous t-PRK + CXL a reduction of the MRSE of 1.74 D and 1.75 D, respectively.

Regarding astigmatism, a significant reduction in the manifest cylinder was achieved in our study changing from a mean preoperative value of −2.9 ± 1.6 and −2.7 ± 1.4 D in simultaneous and sequential groups, respectively, to a 12-month postoperative value of −1.2 ± 0.76 and −0.9 ± 0.65 D in both groups, respectively. Similarly, Sakla et al. [5] reported a decrease of refractive astigmatism from the mean value of −2.77 ± 1.47 D to −0.98 ± 0.76 D. Shaheen et al. [18] demonstrated a change in the manifest astigmatism from a mean preoperative value of −2.79 ± 1.82 D to a 12-month postoperative value of −1.06 ± 0.92 D.

The difficulty in obtaining an accurate value of manifest refractive cylinder in keratoconus eyes may have played an important role on this level of astigmatic undercorrection. Likewise, the biomechanical response of the cornea to the laser ablation and epithelial remodeling may be also significant factors contributing to the observed undercorrection in refractive astigmatism. However, such undercorrection is much lower than that reported for other lines of refractive treatment in keratoconus, such as intracorneal ring segments [22].

Although outcomes predictability is assumed to be much better in sequential approach due to limited precision of manifest refraction in keratoconus before CXL and also due to some expected topographical and refractive changes after CXL procedure [18], our results demonstrated no significant difference in the results of both techniques. However, the repetition of the patient's refraction at least 3 times with a minimum of 1 cycloplegic refraction before the laser procedure is recommended to determine the adequate surgical planning for such cases.

### 4.2. Corneal Topographic Changes

As regards corneal topographical changes occurring after surgery, our results noted a significant decrease in *K*1, *K*2, *K*max, and *Q* values at 6 months after surgery (*P* < 0.001) in both groups, with no additional significant reduction of these parameters during the remaining follow-up (Table 4).

Significant changes were detected in the ISV, *R* min, and KI at 6 months postoperatively with plateau level till the end of follow-up. Comparison of changes occurring in each group during follow-up period revealed no significant difference between the 2 studied groups. Similarly, Shaheen et al. [18] recorded a significant reduction in the corneal indices, the index of surface variation, *R* min, and KI, with improvement in other corneal indices characterizing the regularity of the anterior corneal surface but without reaching statistical significance. The current work also shows that all corneal changes induced are maintained during the follow-up, without signs suggesting a progression of the ectasia as a consequence of the applied laser ablation. In our study, the maximum intended ablation depth did not exceed 50 microns leaving stromal bed at least 350 microns (without the epithelium) and stability of the cornea was observed over a 1-year follow-up. However, future studies should confirm from a biomechanical and structural perspective which criterion is the most adequate for defining the maximum ablation depth in keratoconus after CXL.

Previous studies and basic science research show that surface ablation is better in maintaining the mechanical properties and hysteresis of the cornea compared with other refractive surgery techniques [23–25]. This can be explained by formation of a new fibrocellular membrane in place of the ablated corneal layers that increases the corneal rigidity and acts as a shield helping to prevent further the progression of keratoconus [26]. These structural changes can be associated with the development of haze [23]. However, in our study, there was insignificant corneal haze in the whole sample, which could be attributed to the use of mitomycin C in all the cases after finishing the laser ablation procedure.

### 4.3. Ocular Aberrometric and Mesopic Vision Changes

For both groups the total RMS and HOA were reduced significantly at 6 months after surgery (*P* < 0.001), with additional minimal nonsignificant reductions during the rest of the follow-up (Table 5).

These results are consistent with that obtained in previous studies. Shetty et al. [27] noted significant reduction in total RMS, primary coma, and trefoil RMS at 1 month after simultaneous t-PRK and CXL surgery, with additional significant reductions during the rest of the follow-up. Shaheen et al. [18] recorded significant improvement in aberrometric terms after sequential WFG PRK and CXL that continued during the follow-up.

As far as we know, no previous studies recorded mesopic vision after WFG PRK and CXL in keratoconic patients. Values of contrast sensitivity and glare tests increased significantly 6 months postoperatively till the end of follow-up (*P* < 0.001). Although they improved in sequential group more than simultaneous one, the difference did not reach a significant level. The main reason for the obtained aberrometric and mesopic vision improvement is the reduction of corneal irregularity, which is the main source of HOAs in keratoconus. This effective reduction of corneal irregularity and HOAs is the main cause explaining the significant increase in CDVA.

### 4.4. Subjective Outcome

To the best of our knowledge this is the first research that studies the subjective outcome of simultaneous and sequential WFG PRK and accelerated CXL in patients with keratoconus.

The results recorded statistically significant improvement in all measured parameters with high patient satisfaction attributed to clarity of vision, reduced glare, and halo and spectacles independence. The results demonstrated no significant difference detected between both studied groups (Table 7).

In conclusion, WFG PRK and CXL is safe and effective option to correct the spherocylindrical error and to minimize the level of HOAs in mild and moderate keratoconus. Simultaneous and sequential procedures seem to be equivalent modalities with no significant difference in the recorded objective and subjective outcomes. However, future studies with larger cohorts of patients and longer follow-up periods are needed to determine the safety, efficacy, and stability of such procedure.

## Figures and Tables

**Table 1:** Demographic information of the population enrolled in the study.

Parameter	Values		*P* ^*∗*^
	G 1	G 2	
Number of patients (eyes)	30 (30)	32 (32)	—
Sex (male : female)	14 : 16	15 : 17	—
Mean age ± SD, years	26.3 ± 4.2	24.8 ± 5.5	0.33
Age range, years	21 to 31	22 to 33	—
Gender (male/female)	6/8	7/6	—
Mean refractive cylinder ± SD, D	−2.9 ± 1.6	−2.7 ± 1.4	0.23
Range of refractive cylinder, D	−1.25 to −4.25	−1.4 to −4.3	—
Mean refractive sphere ± SD,	−2.2 ± 1.82	−1.8 ± 1.56	0.12
Range of refractive sphere, D	−0.50 to −3.75	−0.25 to −3.9	—
MRSE (D)	−3.7 ± 2.4	−3.8 ± 2.6	0.52
*K* max D	49.1 ± 4.1	48.3 ± 5.4	0.34
Thinnest pachymetry, um	466 ± 42	475 ± 49	0.81

*P* ^*∗*^: unpaired *t*-test for comparison between the 2 groups.

D: diopter.

MRSE: mean refractive spherical equivalent.

*K* max: maximum keratometry.

**Table 2:** Pre- and postoperative measured parameters (mean ± SD) of the studied patients in sequential group 6 months after CXL alone.

Parameter	Preop.	Postoperative	*P* value
UDVA	0.95 ± 0.31	0.92 ± 0.37	0.42
CDVA	0.29 ± 0.22	0.28 ± 0.19	0.52
Sphere (D)	−1.8 ± 1.56	−1.73 ± 1.92	0.35
Cylinder (D)	−2.7 ± 1.4	−2.6 ± 1.5	0.24
MRSE (D)	−3.7 ± 2.4	−3.6 ± 2.4	0.33
*K*1 (D)	42.9 ± 1.91	42.1 ± 1.93	0.11
*K*2 (D)	45.4 ± 1.83	44.9 ± 1.83	0.09
*K* max (D)	48.3 ± 5.4	46.1 ± 5.6	**0.05**
MCT	475 ± 49	473 ± 53	0.74

Significant *P* values are bolded.

UDVA: uncorrected distant visual acuity.

CDVA: corrected distant visual acuity.

*K* max: maximum keratometry.

MCT: minimum corneal thickness.

D: diopter.

**Table 3:** Preoperative and postoperative visual and refractive parameters (mean ± SD) in the 2 studied groups 6 months and 12 months following the full procedure.

Measure	Group	Preoperative measure	6 months	12 months	*P*; repeated measures ANOVA	*P*; preoperative versus 6 m	*P*; preoperative versus 12 m	*P*; 6 m versus 12 m
UDVA (logMAR)	G1	0.92 ± 0.33	0.19 ± 0.9	0.18 ± 0.06	**<0.001**	**<0.001**	**<0.001**	0.66
	G2	0.95 ± 0.31	0.14 ± 0.11	0.13 ± 0.08	**<0.001**	**<0.001**	**<0.001**	0.45
	*P* ^*∗*^	0.35	0.08	0.07	—	—	—	—

CDVA (logMAR)	G1	0.32 ± 0.25	0.14 ± 0.07	0.13 ± 0.08	**<0.001**	**<0.001**	**<0.001**	0.86
	G2	0.29 ± 0.22	0.09 ± 0.06	0.09 ± 0.05	**<0.001**	**<0.001**	**<0.001**	0.91
	*P* ^*∗*^	0.41	0.13	0.06	—	—	—	—

Refractive sphere (D)	G1	−2.2 ± 1.82	−0.55 ± 0.82	−0.53 ± 0.79	**<0.001**	**<0.001**	**<0.001**	0.93
	G2	−1.8 ± 1.56	−0.46 ± 0.66	−0.42 ± 0.63	**<0.001**	**<0.001**	**<0.001**	0.12
	*P* ^*∗*^	0.61	0.42	0.33	—	—	—	—

Refractive cylinder (D)	G1	−2.9 ± 1.6	−1.2 ± 0.79	−1.2 ± 0.76	**<0.001**	**<0.001**	**<0.001**	0.98
	G2	−2.7 ± 1.4	−0.9 ± 0.66	−0.9 ± 0.65	**<0.001**	**<0.001**	**<0.001**	0.87
	*P* ^*∗*^	0.53	0.42	0.37	—	—	—	—

MRSE (D)	G1	−3.7 ± 2.4	−1.4 ± 0.72	−1.3 ± 0.79	**<0.001**	**<0.001**	**<0.001**	0.67
	G2	−3.8 ± 2.6	−1.1 ± 0.71	−1.00 ± 0.68	**<0.001**	**<0.001**	**<0.001**	0.88
	*P* ^*∗*^	0.77	0.46	0.34	—	—	—	—

*P* ^*∗*^: unpaired *t*-test for comparison between the 2 groups.

Significant *P* values are bolded.

UDVA: uncorrected distant visual acuity.

CDVA: corrected distant visual acuity.

*K* max: maximum keratometry.

MRSE: mean refractive spherical equivalent.

D: diopter.

**Table 4:** Preoperative and postoperative topographical (Pentacam) parameters (mean ± SD) in the 2 studied groups 6 months and 12 months following the full procedure.

Measure	Group	Preoperative measure	6 months	12 months	*P*; repeated measure ANOVA	*P*; preoperative versus 6 m	*P*; preoperative versus 12 m	*P*; 6 m versus 12 m
*K*1 (D)	G1	43.3 ± 1.77	41.1 ± 1.33	41.0 ± 1.31	**<0.001**	**<0.001**	**<0.001**	0.91
	G2	42.9 ± 1.91	40.8 ± 1.39	40.9 ± 1.40	**<0.001**	**<0.001**	**<0.001**	0.88
	*P* ^**∗**^	0.23	0.55	0.31	—	—	—	—

*K*2 (D)	G1	45.9 ± 2.45	42.5 ± 1.45	42.1 ± 1.44	**<0.001**	**<0.001**	**<0.001**	0.92
	G2	45.4 ± 1.83	41.7 ± 1.62	41.8 ± 1.63	**<0.001**	**<0.001**	**<0.001**	0.79
	*P* ^**∗**^	0.11	0.37	0.42	—	—	—	—

*K*max (D)	G1	49.1 ± 4.1	46.2 ± 4.2	45.8 ± 4.4	**<0.001**	**<0.001**	**<0.001**	0.54
	G2	48.3 ± 5.4	44.9 ± 5.5	45.2 ± 5.3	**<0.001**	**<0.001**	**<0.001**	0.78
	*P* ^**∗**^	0.88	0.71	0.64	—	—	—	—

*Q*	G1	−0.47 ± 0.39	−0.29 ± 0.31	−0.28 ± 0.33	**<0.001**	**<0.001**	**<0.001**	0.77
	G2	−0.45 ± 0.41	−0.27 ± 0.33	−0.27 ± 0.34	**<0.001**	**<0.001**	**<0.001**	0.64
	*P* ^**∗**^	0.76	0.44	0.54	—	—	—	—

MCT	G1	466 ± 42	418 ± 37	421 ± 32	**<0.001**	**<0.001**	**<0.001**	0.43
	G2	475 ± 49	431 ± 39	433 ± 40	**<0.001**	**<0.001**	**<0.001**	0.71
	*P* ^**∗**^	0.22	0.09	0.12	—	—	—	—

ISV	G1	44.8 ± 26.8	39.2 ± 23.9	39.3 ± 23.8	**0.001**	**0.003**	**0.003**	0.91
	G2	43.9 ± 25.8	38.9 ± 22.1	38.8 ± 22.3	**0.001**	**0.004**	**0.004**	0.65
	*P* ^**∗**^	0.48	0.94	0.76	—	—	—	—

IVA	G1	0.47 ± 0.33	0.44 ± 0.28	0.45 ± 0.29	0.33	0.41	0.45	0.39
	G2	0.43 ± 0.30	0.41 ± 0.29	0.42 ± 0.29	0.27	0.38	0.41	0.32
	*P* ^**∗**^	0.55	0.68	0.73	—	—	—	—

IHA	G1	19.8 ± 11.2	18.3 ± 11.8	18.2 ± 12.1	0.77	0.69	0.71	0.59
	G2	18.3 ± 10.5	17.6 ± 10.6	17.5 ± 10.3	0.67	0.61	0.64	0.93
	*P* ^**∗**^	0.71	0.61	0.29	—	—	—	—

*R* min	G1	6.9 ± 0.45	7.4 ± 0.49	7.4 ± 0.49	**0.021**	**0.032**	**0.033**	0.83
	G2	7.1 ± 0.47	7.5 ± 0.51	7.6 ± 0.52	**0.041**	**0.049**	**0.050**	0.57
	*P* ^**∗**^	0.38	0.77	0.22	—	—	—	—

KI	G1	1.1 ± 0.09	1.07 ± 0.1	1.07 ± 0.1	**0.009**	**0.008**	**0.008**	0.89
	G2	1.2 ± 0.1	1.09 ± 0.1	1.09 ± 0.1	**0.008**	**0.006**	**0.006**	0.92
	*P* ^**∗**^	0.99	0.86	0.81	—	—	—	—

*P* ^*∗*^: unpaired *t*-test for comparison between the 2 groups.

Significant *P* values are bolded.

*K*max: maximum keratometry.

*Q*: corneal asphericity.

MCT: minimum corneal thickness.

ISV: index of surface variance.

IVA: index of vertical asymmetry.

IHA: index of height asymmetry.

*R* min: minimum radius of curvature.

KI: keratoconus index.

**Table 5:** Preoperative and postoperative aberrometric parameters and mesopic vision testing results (mean ± SD) in the 2 studied groups 6 months and 12 months following the full procedure.

Measure	Group	Preoperative measure	6 months	12 months	*P*; repeated measure ANOVA	*P*; preoperative versus 6 m	*P*; preoperative versus 12 m	*P*; 6 m versus 12 m
Total RMS	G1	5.1 ± 1.61	2.1 ± 0.71	2.0 ± 0.73	**<0.001**	**<0.001**	**<0.001**	0.49
	G2	5.0 ± 1.72	1.89 ± 0.76	1.8 ± 0.77	**<0.001**	**<0.001**	**<0.001**	0.35
	*P* ^**∗**^	0.76	0.11	0.12	—	—	—	—

HOA RMS	G1	1.2 ± 0.79	0.77 ± 0.33	0.76 ± 0.31	**<0.001**	**<0.001**	**<0.001**	0.88
	G2	1.1 ± 0.77	0.71 ± 0.41	0.69 ± 0.39	**<0.001**	**<0.001**	**<0.001**	0.81
	*P* ^**∗**^	0.45	0.09	0.25	—	—	—	—

Contrast sensitivity (%)	G1	54.2 ± 6.8	70.1 ± 8.3	73.1 ± 10.1	**<0.001**	**<0.001**	**<0.001**	0.08
	G2	56.6 ± 7.8	71.7 ± 8.1	74.8 ± 9.5	**<0.001**	**<0.001**	**<0.001**	0.07
	*P* ^**∗**^	0.24	0.56	0.08	—	—	—	—

Glare (%)	G1	34.1 ± 10.3	49.8 ± 9.7	51.1 ± 8.8	**<0.001**	**<0.001**	**<0.001**	0.11
	G2	33.8 ± 9.4	47.8 ± 9.6	50.4 ± 8.9	**<0.001**	**<0.001**	**<0.001**	0.06
	*P* ^**∗**^	0.69	0.44	0.12	—	—	—	—

*P* ^**∗**^: unpaired *t*-test for comparison between the 2 groups.

Significant *P* values are bolded.

Total RMS: total root mean square.

HOA RMS: high order aberration root mean square.

**Table 6:** Comparison of changes over 12-month period in visual, refractive, aberrometric, and topographical parameters between the 2 studied groups.

Parameters	G1	G2	*P* ^*∗*^ value
ΔUDVA (logMAR)	−0.74 ± 0.31	−0.83 ± 0.38	0.33
ΔCDVA (logMAR)	−0.19 ± 0.16	−0.24 ± 0.15	0.59
ΔRefractive sphere (D)	1.67 ± 1.16	1.48 ± 1.22	0.37
ΔRefractive cylinder (D)	1.7 ± 1.11	1.9 ± 1.38	0.71
ΔMRSE (D)	2.4 ± 1.91	3.00 ± 1.82	0.53
ΔTotal RMS	−3.1 ± 1.2	−3.2 ± 1.3	0.83
ΔHOA RMS	−0.44 ± 0.22	−0.41 ± 0.31	0.64
ΔContrast sensitivity (%)	18.9 ± 7.4	20.2 ± 7.9	0.56
ΔGlare (%)	17 ± 6.8	19.6 ± 7.3	0.22
Δ*K*1 (D)	−2.3 ± 1.6	−2.00 ± 1.7	0.61
Δ*K*2 (D)	−3.8 ± 1.7	−3.6 ± 1.8	0.46
Δ*K*max (D)	−3.5 ± 2.1	−3.1 ± 1.9	0.89
ΔMCT	−45 ± 23.1	−42 ± 21.9	0.16

*P* ^*∗*^: unpaired *t*-test for comparison between the 2 groups.

UDVA: uncorrected distant visual acuity.

CDVA: corrected distant visual acuity.

*K* max: maximum keratometry.

MRSE: mean refractive spherical equivalent.

Total RMS: total root mean square.

HOA RMS: high order aberration root mean square.

D: diopter.

**Table 7:** Patient self-assessed mean subjective ratings preoperatively and 12 months postoperatively in both groups (mean ± SD).

Parameter	Group	Preop.	12 months postop.	*P* ^+^ value
Clarity of vision	G1	2.89 ± 0.43	4.41 ± 0.46	**<0.001**
	G2	2.77 ± 0.49	4.46 ± 0.51	**<0.001**
	*P* ^*∗*^	0.23	0.44	—

Patient satisfaction	G1	2.22 ± 0.41	4.71 ± 0.41	**<0.001**
	G2	2.41 ± 0.39	4.81 ± 0.22	**<0.001**
	*P* ^*∗*^	0.38	0.67	—

Glare	G1	3.66 ± 0.43	2.01 ± 0.99	**<0.001**
	G2	3.82 ± 0.41	2.21 ± 1.1	**<0.001**
	*P* ^*∗*^	0.74	0.11	—

Halo	G1	3.88 ± 0.47	2.23 ± 0.37	**<0.001**
	G2	3.91 ± 0.39	2.4 ± 0.25	**<0.001**
	*P* ^*∗*^	0.81	0.61	—

Far spectacle dependence	G1	4.8 ± 0.02	0.76 ± 0.61	**<0.001**
	G2	5.00 ± 0.00	0.81 ± 0.66	**<0.001**
	*P* ^*∗*^	0.31	0.46	—

*P* ^+^: paired *t*-test for comparison between preoperative and postoperative values of the same group.

*P* ^*∗*^: unpaired *t*-test for comparison between the 2 groups.

Significant *P* values are bolded.
